# Supplementary material for: Are carbon emissions trading and green financial instruments synergistic? -Comprehensive quantitative research based on content analysis
Source: PLoS One. 2024 Mar 7;19(3):e0298601. doi: 10.1371/journal.pone.0298601 (PMC10919601; doi:10.1371/journal.pone.0298601)
Supplement: S1 Data — (ZIP) [file pone.0298601.s001.zip › Data/地级市Z_F工作报告.zip!/╝¬┴╓/░╫│╟╩╨/└┤╫╘╣1⁄2╓┌║┼í╛┬φ┐╦╩2╛▌═°í┐_2003.docx]

各位代表：

　　我代表白城市人民政府，向大会报告政府工作，请予审议，并请市政协各位委员提出意见。

　　一、2002年工作回顾

　　2002年，在市委的领导下，在市人大依法监督和市政协民主监督下，我们努力践行"三个代表"重要思想，团结带领全市人民开拓进取，与时俱进，拼搏奋斗，在2001年实现省内综合实力排序升一位的基础上，各项工作都取得了新成绩，完成了市二届人大四次会议确定的政府各项任务。

　　----国民经济持续快速增长。全市国内生产总值完成105亿元，同比增长15.7%（预计数，按可比口径计算，下同），其中，第一产业增加值36.6亿元，同比增长15.1%；第二产业增加值34.7亿元，同比增长18.5%；第三产业增加值33.7亿元，同比增长13.7%。全口径财政收入72170万元，同比增长17.4%。固定资产投资36亿元，同比增长27.7%。城镇居民人均可支配收入5100元，农民人均纯收入2060元，同比分别增长18.4%和60.9%。

　　坚持以提速增效为重点，努力提高工业运行质量，速度和效益实现较快增长。规模以上工业总产值37.2亿元，同比增长19%；地方工业利润1.89亿元，同比增长33.1%。工业销售收入36.1亿元，同比增长29.4%，工业产品产销率累计完成96%，提高1.2个百分点。开发改造取得新进展，开工项目80项，其中建成50项，完成投资5.1亿元，同比增长19.7%。开发新产品158种，产值率达19%。民营经济和乡镇企业总产值56.7亿元，同比增长17%。

　　立足提高农业效益，着力调整优化结构，农村经济全面发展。农业总产值67.2亿元，同比增长24.4%；种植业粮经饲比例为4：4：2；粮食产量37.5亿斤，同比增长44.5%，辣椒、蓖麻、花生、烤烟等特色经济作物已形成规模。发展万元田棚2.9万户，总户数达11万户。落实股份合作牧业户5500个，实现产值1.08亿元；草原建设、畜禽品种改良和舍饲水平有了新提高。超额完成了春季60万亩生态林（草）会战和秋季造林任务，造林55.9万亩，营造生态草30万亩。植桑2690亩，完成计划的119%。嫩江、洮儿河堤防建设和"引霍入向"等水利设施工程完成了阶段任务，新打农田井5744眼，新增节水灌溉面积10.3万亩，人工增雨能力明显增强。农业机械化和科技示范园建设迈出了新步伐。举办了农业科技那达慕大会。在长春农博会上，我市又有42种产品被评为名牌产品，国家和省名牌产品总数达263种。农产品已远销日本、新加坡、越南、斯里兰卡等十几个国家。全市签订农产品订单460万亩，占农作物播种面积的46.3%。农村各类中介服务组织发展到375个。加强了扶贫开发工作，9.1万农村贫困人口实现脱贫。

　　抓住入世机遇，积极发展商贸经济，不断开拓国内外市场，流通领域日趋活跃。全市社会消费品零售总额49.5亿元，同比增长9.8%。外贸进出口额4100万美元，同比增长23.3%。销售粮食60万吨；开发了"向海"牌卷烟，地方品牌卷烟销售5000大箱。市场建设步伐加快，聚龙建材城、瑞光商贸城、阳光广场、吉鹤商都等投入运营，吉鹤蔬菜批发市场主体工程基本完工，通榆中国关东马市、中国o镇赉大牲畜交易市场相继开市；举办了中国o北方白鹅产品交易会、中国o白城红干椒贸易招商洽谈会、中国o洮南杂粮杂豆展洽会；组织参加了全国跨国零售集团商品定货会、吉林省首届消费精品展洽会和第四届吉菜美食节等商贸交流活动，市场领域不断拓宽。超市、连锁、仓储、配送等新型商业迅速发展。

　　全面落实重点项目建设责任制，积极走出去争取和引进项目，项目建设步伐明显加快。规划千万元以上重点项目131项，今年完成投资8.78亿元。其中，派克高压阻尼点火线总成等29项续建项目全面启动；镇赉华海油田、多邦药业等37项新开项目已开工建设；规划储备了向日葵综合开发、中加燕麦等65个项目，项目规划和建设水平有了新提高。

　　----改革实现新突破。企业改革进一步深化，白城中药厂、大安石油机械厂等企业重组整合取得了实质性进展，一批困难企业通过购并重组恢复了活力。企业法人治理结构得到进一步规范，破产重组工作进展顺利。完成了市县乡机构改革。积极探索事业单位改革，市公路工程处、水利工程处等单位完成了改制工作。机关事业单位基本医疗保险改革启动实施。小城镇综合改革取得新进展，安广镇、岭下镇、瞻榆镇被评为省先进镇。科技教育卫生体制改革、农村税费改革、土地使用权流转等改革全面推进。

　　----对外开放成果显著。积极拓展双招双引渠道，借助外力加快发展。在北京和深圳成功举办了中国o白城经贸项目发布会、洽谈会，在长春召开了民营经济经贸洽谈会，积极参加省组织的日本东京中国贸易展览会、澳大利亚东华节吉林省展洽会，组织参加“青洽会”、"乌洽会"、"厦洽会"、香港o吉林友谊周等招商引资活动，收到了明显成效。全市招商引资项目550项，到位资金33.6亿元，同比增长35%。组织群众走出去，发展劳务经济，劳务输出32万人，实现收入10亿元。组团赴北京、长春、青海和新疆等地开展招贤引智活动，共引进国内外人才196人。

　　大力培育旅游产业，加快发展旅游经济。成功举办了中国o白城第三届生态旅游节，在北京、深圳、长春等地分别举办了新闻发布会；在大连、沈阳、哈尔滨和吉林等城市开展了旅游产品促销活动。多渠道筹资2亿多元，建设完善了查干浩特、向海、莫莫格等景区，开辟了31个景区景点，开发了20多条旅游线路。与兴安盟、阿尔山市签订了旅游开发联动促销协议，打造品牌，整合旅游资源。全市接待国内外游客70万人（次），旅游收入1.98亿元，同比分别增长42%和18%。

　　推进开发区建设，增强对全市经济的拉动力。白城经济开发区国内生产总值2.15亿元，同比增长41%，财政收入1100万元，同比增长83.3%。民营经济开发区引进项目14个，到位资金1亿元。查干浩特旅游开发区完成了环岛油路、园区绿化等33项工程。大安经济开发区完成了小油田开发、骨素明胶二期改造等工程。

　　----基础设施建设步伐加快。把握机遇，运用市场机制开发城市、建设城市、管理城市、经营城市，城市开发建设管理总体战取得新突破。在全面完成年初确定的142件实事的同时，完成了22项新增工程，总投资28.5亿元，其中市区投资16.8亿元。市区和县城新建、改造、维修、硬化道路158条，80.3万平方米；新建住宅116万平方米；新增绿地面积440万平方米。蓝天工程综合整治了566台耗能高、污染大的锅炉、茶炉、燃煤灶，拆掉大小烟囱104个，大气总悬浮微粒等项指标同比下降10%，市区和县城大气环境质量分别保持在国家二级、三级标准内。完成了市区主要街路两侧135座建筑物、牌匾橱窗的亮化和白洮一级路、文化西路、幸福北街路灯工程建设。总体战的其它各项工程也按计划如期完工。加强了城市管理，全市卫生状况、交通秩序、城市面貌有了新的改观。强化城市土地资本运营，建立健全了土地收购储备制度，实现土地收益2528万元。公路建设取得了可喜成绩，长白一级路白城至大安段全线贯通，完成路网工程254公里，实现了乡乡通油路。信息和通讯设施进一步完善。

　　----各项社会事业全面发展。努力推进科技创新，取得各类科技成果20项，科技示范园区建设步伐加快。整合教育资源，成立了白城行政管理学院、白城师范学院经济管理学院、吉林农机研究院白城分院；与长春工业大学签订了联合创办白城工学院协议。基础教育水平有了新提高，高考本科进线率居全省第二位，有12人考入清华和北京大学，创历年最好水平。文化体育事业蓬勃发展，成功举办了首届文化节和体育那达慕大会；"两节"期间组织各类文艺演出500余场（次）；我市运动员在北京国际马拉松5公里赛上获2枚金牌，在省第十四届运动会上获35枚金牌。广播电视获省优秀节目一等奖14项，获国家级三等奖2项，广电大厦已建成投入使用。卫生体制改革不断深化，全市二级以上医院统一实行了药品集中招标采购制度。计划生育工作受到国家人事部、计生委的表彰。民族、宗教、侨务、地方志、老龄、妇女儿童、残联、红十字会等事业有了新发展，国防后备力量建设、双拥、人防、防震减灾等工作取得了新成绩。

　　----城乡社会保持稳定。加强了社会保障工作，全民所有制职工省级统筹养老保险覆盖面为99%，下岗进站职工基本生活费发放率和代缴社会保险费率为100%，8.39万人纳入了低保范围；积极扩大就业和再就业，开发就业岗位3.9万个，2.7万名下岗失业人员实现了再就业。落实了信访领导责任制，认真接待群众来信来访，努力化解和妥善处理各种社会矛盾。整顿规范市场经济秩序，重点整治了农资、药品、烟草、建筑、文化等市场和加油站。加强安全生产责任落实和专项整治，确保了生产安全。严厉打击各种严重刑事犯罪及“法轮功”等邪教组织的违法活动，大力加强社会治安综合治理，保持了政治安定和社会稳定。

　　----民主法制和政府自身建设进一步加强。完善了向人大报告工作和向政协通报工作制度，认真执行人大决议和决定，定期向人大常委会报告工作，积极支持政协及各民主党派、工商联参政议政，邀请人大代表、政协委员视察重点工作进展情况，自觉接受人大依法监督和政协民主监督。认真办理人大代表建议、议案和政协委员提案，共承办人大代表建议和议案29件、政协委员提案116件，全部办理完毕。认真组织行政执法检查，做好行政复议工作，促进依法行政。深入开展普法教育，增强了干部群众的法律意识和依法办事的自觉性。积极推?四制"，加大行政审批制度改革力度，对行政审批项目进行了全面清理、核查和审定，颁布了白城市人民政府第9号令，停止执行282项行政审批项目。进一步扩大政务公开内容，强化政务公开手段，在政府网站公布了部门审批事项；在电视台开辟了"部门领导谈承诺"栏目；在《白城日报》刊登了市直22个部门的服务承诺。加强了市长和县（市、区）长公开电话工作，群众所反映的问题基本得到妥善解决。有效地开展了经济监督和领导干部经济责任审计。认真落实领导干部廉洁自律的各项规定，实行“一岗双责”责任制，继续纠正部门和行业不正之风，加大案件查办力度，廉政建设取得了新进展。

　　2002年所取得的成绩，是全市各级干部和广大人民群众解放思想，开拓进取，团结奋斗的结果；是社会各界、方方面面大力支持的结果。我代表市政府向全市工人、农民、知识分子、各阶层群众和各级干部，向各级人大、政协、民主党派、工商联、人民团体及各界爱国人士，向驻白部队、武警官兵、公安司法干警和中省直驻白单位，向对我们的工作给予关心和帮助的离退休老领导、老同志表示衷心的感谢和崇高的敬意！

　　同时，我们也清醒地看到全市经济社会发展中存在的问题，主要是：工业规模小，结构不尽合理，部分企业生产经营困难；农业产业化层次低，综合效益不高，一些地方因受灾较重，农民收入增长缓慢；第三产业发展不平衡，现代服务业发展水平相对较低；财政收支矛盾仍较突出；就业和再就业压力增大，城乡部分弱势群体生活较困难，社会不稳定因素依然存在；政府职能转变还有差距，经济发展环境需要进一步改善。对这些问题，我们要高度重视，在今后的工作中切实加以改进和解决。

　　二、2003年经济社会发展目标、任务

　　2003年是全面贯彻落实党的十六大精神，加快白城跨越式发展，推进小康社会建设的重要一年。我们要按照发展要有新思路，改革要有新突破，开放要有新局面，各项工作要有新举措的要求，审时度势，抢抓机遇，在国内外市场竞争中营造优势；奋发图强，争创一流，在推进整体升位中实现跨越；拼搏奋斗，克难制胜，在全面建设小康社会中加快发展。

　　市政府工作总体思路是：认真贯彻党的十六大和市委三届三次全会精神，以"三个代表"重要思想为指导，以加快推进全面建设小康社会进程为目标，以实现"三步走、三基本"规划为主要任务，实施开放带动、科教兴市、县域突破、人才兴业战略，坚持更新观念争上游、负重前进加压力、改革创新找差距、跨越发展升位次方针，加快推进工业化、信息化、城市化、经济国际化进程，建设汽配纺织医药城、区域商贸中心城、生态环保旅游城。突出抓好项目建设，集聚总量增强实力。在抓好物质文明建设的同时，加强政治文明和精神文明建设，推进国民经济持续快速健康发展和社会全面进步。

　　主要预期目标：全市国内生产总值119亿元，按可比口径同比增长14.4%；固定资产投资41.4亿元，同比增长15%；全口径财政收入80758万元，同比增长12%；城镇居民人均可支配收入5600元，同比增长10%；农民人均纯收入2300元，同比增长12%；人口自然增长率控制在6‰以内。力争使城乡居民生活质量和思想道德、科学文化、健康素质有新的提高；生态环境得到改善，增强可持续发展能力。

　　为了完成全年目标，主要抓好以下八项工作：

　　（一）努力提高工业经济效益，推进工业化进程

　　继续实施"兴工富市"战略，积极探索走新型工业化道路的有效途径，以信息化带动工业化，以工业化促进信息化，培育壮大支柱和优势产业，加快产业创新和升级，努力构建以传统支柱产业为支撑、以高新技术产业为先导、以新兴产业为后续的工业体系。规模以上工业总产值同比增长15%。

　　抓住入世机遇，走"融入"式发展之路。围绕汽车配套、纺织服装、医药化工等支柱产业和食品、能源、建材等重点行业的骨干企业，本着优势互补原则，采取请进来、走出去的方式，积极寻求战略性投资伙伴，主动与境外跨国公司、大企业、大集团合作重组。吸引和支持国内外企业到我市开发项目、兴办企业，提高工业市场化、国际化水平。发挥规模以上工业市场条件较好、生产潜能大的优势，确保提速增效；对规模以下企业，采取分工协作、扩散配套零部件产品等方式扶持其发展。鼓励和扶持有条件的乡镇、村办企业迁址进城（镇），兴办农产品加工型企业，推动工业经济发展。

　　加快企业技术进步，促进产业升级。全市技术改造投资要完成6亿元，同比增长17%。全力争取国债贴息技改项目，实施好支柱产业和优势行业的30个重点技改项目，提高技术水平和竞争能力。坚持以高新技术改造传统产业，加大新产品开发力度，抓好160种新产品和11种高新技术产品的开发。积极开发建设环保节能、清洁能源等新兴工业。

　　大力开拓市场，提高竞争能力。按照国际市场需求、供货方式和通行规则，调整营销策略。组织企业走出去，开展各种形式的经贸交流活动，积极参加各类订货会、洽谈会和招商会，宣传和推销优势、特色产品，进一步扩大市场覆盖面。开展好“企业上网年”活动，充分利用政府网站和中国吉林企业网等现代信息手段，推进电子商务、网上销售业务，努力使地方产品进入国际、国内大型购销网络中，拓展市场领域，提高经济效益。

　　（二）以增加农民收入为核心，加快农村经济发展

　　坚持用工业化思维谋划农业发展，优化农村经济结构，大力发展生产规模相对集中、品牌特色相对统一、加工服务相对配套的园区农业，推进农业产业化，切实转变增长方式。

　　根据市场需求，立足我市实际，加快农业结构调整。在种植业结构调整上，力争实现"一减一增一稳"，继续减少普通粮食，特别是普通玉米种植面积，增加经济效益好、抗旱能力强的杂粮杂豆、无公害蔬菜、绿色食品和饲草、饲料作物面积，稳定旱涝保收的水稻面积。在农业产业结构调整上，大力发展畜牧业，增加畜牧业在全市农业总产值中的比重，逐步使畜牧业成为我市农村经济的主导产业。抓好农副产品加工和贸易，促进其转化增值。在农业组织结构调整上，加强专业合作社、公司加农户、专业协会、股份合作、民营庄园等新型农村经济组织建设，提高农业的组织化程度和分工协作的产业化水平。

　　充分发挥比较优势，推进万元田棚建设，力争在上标准、扩规模、增效益上实现突破。搞好科学规划和合理布局，实行连片开发，向基地规模化、生产专业化、管理标准化、经营产业化方向发展，构筑数村一品、数乡一业的园区型万元田棚格局。因地制宜，筛选有市场、效益好的品种和项目，在政策、资金、技术等方面给予大力扶持。要提高产品档次，增加科技含量，突出特色，争创名牌，扩大市场份额。万元田棚要发展到13.6万户，占农村总户数的50%。

　　以市场为导向，推进股份合作牧业经济，加快发展畜牧业。研究制定股份合作牧业经济管理办法，加大投入，扩大规模，建立有序的运行机制，使股份合作牧业经济走上制度化、规范化和市场化轨道。抓好精品牧业小区建设和培育牧业强乡、强村工作，在规划设计、政策扶持、技术指导、跟踪服务等方面强化措施，提高饲养质量和水平。搞好无规定动物疫病区建设，加快建设畜产品生产、加工、销售安全体系。抓好规模养殖，有计划扶持养殖大户，充分发挥示范带动作用，提高畜牧业集约化经营水平。

　　加快生态示范区工程建设，努力改善农业生产条件和生态环境。重点抓好"三环一路一网"绿化和退耕还林、三北四期防护林、湿地及野生动植物保护、生态草工程建设。完成造林30万亩，营造生态草30万亩，建设标准化草场30万亩，发展林业和草业经济。抓好江河堤防水库除险加固、抗旱水源及节水灌溉工程、灌区续建配套工程建设，做好人工增雨工作，积极利用地上水，合理开发地下水，科学利用水资源。组织好"引霍入向"，月亮湖蓄滞洪区双向闸，向海、团结、创业、兴隆、胜利水库除险加固工程等重点水利项目建设。本着集中开发、集约经营的原则，积极开发桑蚕业，植桑1.5万亩，为今后规模开发奠定良好基础。全面落实扶贫攻坚计划，积极争取国家和省扶贫资金，抓好扶贫开发移民，千方百计增加灾区和贫困地区农民收入，加快脱贫致富进程。

　　（三）充分发挥区位优势，大力发展第三产业

　　加强市场建设和管理。重点培育白城经济开发区建筑装饰装潢材料、洮北红干椒、通榆和镇赉大牲畜交易、洮南杂粮杂豆、大安白鹅等大型市场，抓好市场设施建设，强化管理，扩大经营规模，提高运作能力。进一步完善市区通讯器材、卫生陶瓷、汽车摩托车配件、副食品等12条零售专业街。加强对各类市场的规范和管理，维护公平、有序的市场秩序。

　　努力搞活内外贸易。进一步扩大特色产品、绿色食品和地方产品的销售，加强粮食、地方品牌卷烟等产品的外销。社会消费品零售总额达到54.5亿元，同比增长10%。落实好国家鼓励出口的各项政策措施，在巩固欧美和东南亚等主要市场的同时，开发新的出口市场，着力培育出口支柱产业、重点企业的自营出口能力，扩大出口规模。外贸出口总额完成2000万美元，同比增长15%。积极发展信息、金融、保险、中介、社区等新兴服务业，推广连锁经营、电子商务、分销代理、超市等现代流通业，不断满足人民群众生活需求。大力引进名企、名店、名品，吸引国内外经纪人进入，同时创造条件到省外、国外经商办店，构筑大商贸、大流通的格局。

　　加快发展生态旅游业。按照全市旅游总体规划，重点抓好向海、莫莫格、查干浩特和环市区等景区景点完善和建设，增加景点的文化内涵，加快开发旅游产品。办好第四届生态旅游节和旅游产品展销会，组织?quot;鹤城之旅"主题活动，全方位开展宣传、推介和促销，打响旅游品牌，把季节性旅游发展为全年性旅游，逐步使白城旅游融入国内外旅游大市场。加强旅游行业管理，完善服务设施，搞好旅游从业人员培训，高质量地开展接待服务，提高旅游业发展水平。

　　（四）加快项目建设步伐，增强综合经济实力

　　围绕项目总体规划，扎扎实实做好项目工作。在优势产业项目建设上，从发展规模经济、提升产业层次出发，加快支柱产业和重点行业项目开发建设。充分利用风能资源，发展风力发电项目，全力抓好列入省"十五"风电开发计划的通榆风电三期续建项目，洮北、洮南、镇赉风电新开工项目；积极支持石油资源的开发利用，建立吉林省西部能源基地。在农业产业化项目建设上，组织好桑蚕、草业、芦苇、烤烟、饲料、蓖麻、草原红牛等项目的开发和建设，通过产业化带动农村经济发展。在基础设施项目建设上，重点抓好城市污水垃圾处理、道路、园林等市政设施项目建设；加快207线镇赉至坦途一级路、市区路网等道路建设，进一步改善城乡环境。在社会事业项目建设上，围绕发展教育、文化、卫生、广电等事业积极运作项目，争取国家和省的支持。在高新技术产业项目开发上，加快草原红牛胚胎生物工程等项目启动，积极创办高新技术企业，推动产业结构优化升级。

　　（五）深化各项改革，全方位扩大开放

　　坚持以推进体制和机制创新为重点，继续深化企业改革。加快重组整合，对尚未退出国有系列的中小企业，分类指导，能出售的出售，能重组的重组；对不能重组的，在妥善安置职工的前提下依法破产；对已到租期的国有企业，鼓励支持承租人购并或改制为股份制企业；国有控股企业中的国有股及资产要尽快退出，对已完成公司制改造企业的国有股实行减持式退出；鼓励支持没有国有股权企业优化股本结构，实行少数人持大股，增强企业发展动力。完善法人治理结构，推进企业制度创新。按照管资产和管人、管事相结合的原则，积极探索有效的国有资产管理体制和方式。开展好年薪制、期股期权试点。按照依法、自愿、有偿的原则，搞活土地使用权流转，促进土地规模经营。深化粮食流通体制改革，努力搞活粮食营销。积极稳妥推进农村税费改革，建立有效的农民负担监督管理机制，切实减轻农民负担。深化财政管理改革，完美财政监督机制。大力发展非公有制经济，全面改善民营经济的体制环境、政策环境和服务环境。组建民营企业协会，提高民营经济组织化程度。全市个体工商户和私营企业分别增长13%和12%。加快小城镇综合改革与发展，重点培育发展16个中心集镇，其中建设好3个省级中心镇、7个省级"十强镇"、6个市级试点镇。积极推进户籍、土地、投融资、社会保障等方面改革，大力发展特色经济，完善基础设施建设，使其在经济增长、吸纳农村剩余劳动力、促进农业产业化和城镇建设方面发挥支撑、示范和辐射带动作用。深化住房制度改革，建立住房公积金制度。继续抓好事业单位体制改革和机关事业单位基本医疗保险改革，积极推进金融、科技、文化、教育、卫生等方面改革，进一步增强发展活力。

　　坚持"双招双引"基本市策和"四个走出去"策略，在更大范围、更广泛领域推进对外开放，多层次、全方位拓展引资引智渠道。招商引资完成38.6亿元，同比增长15%。积极组织参加各种招商活动，发挥企业的主体作用，力争在引进项目上取得新进展，在引进国内外大公司、大集团上实现新突破。积极探索多种利用外资和合作方式，开展项目融资、股权融资、债券融资，推行地域招商、代理招商、网上招商等招商方式，扩大招商引资成果。加快发展劳务经济，劳务输出30万人，实现收入12亿元。完善吸引人才的优惠政策，创新人才培养、引进、流动、使用管理机制和环境。积极引进和培养我市急需的国际商贸、高新技术、信息、旅游等方面的专业人才和企业经营管理人才。建立人才"数据库"和信息网络系统，畅通人才引进渠道。

　　加快开发区建设，使其率先在体制创新、产业升级、扩大开放等方面发挥示范带动作用。白城经济开发区重点抓?五园"建设，生物医药园争取有3户企业投产；尽快启动高新技术园。民营经济开发区继续加快重点项目建设。大安经济开发区组织好农产品深加工项目建设。查干浩特旅游开发区要在体制和机制创新中增强活力，抓好新的旅游项目开发。

　　（六）抓好城市基础设施建设，提高经营城市水平

　　城市是最大的国有资产。要用市场经济的眼光认识城市，增强经营城市意识，对城市资源进行聚集、重组和运营，最大限度地盘活存量，优化增量，做“以城养城、以城建城、以城兴城”，走一条建设市场化、资金筹措多元化、资源利用商品化的城市建设、管理新路子。

　　坚持"一城崛起、县城争辉、中心镇突破"的发展思路，继续在全市范围内开展城市开发建设管理总体战，加大投入，强化管理，提高城市品位。城市开发建设计划总投资26.22亿元，其中市区投资13.47亿元，各县（市）投资12.75亿元。市区集中抓好十大工程（139件实事）：一是道路工程。新建、拓宽道路14条，铺装巷道、人行道26条。二是重点基础设施建设工程。实施北部供热站等工程建设；抓紧污水处理厂、污水管网扩建、金辉公铁立交桥和三环路前期工作，力争开工建设。三是住宅建设工程。计划建设住宅58万平方米。四是市区绿化工程。抓好广场、小区、巷道、二环路分车岛绿化和拆墙透绿、立体绿化建设。五是亮化工程。搞好路灯、高大建筑物射灯、临街商店和橱窗霓虹灯建设。六是蓝天工程。对市区二环路、出入口规定范围内的锅炉、炉灶限期进行达标整治。七是环卫设施建设工程。新建6座垃圾中转站和10座标准公厕。八是公共客运交通建设工程。新增40 台公交车。九是社会公益事业建设工程。完成政务服务中心大厦等21项新建、扩建、续建工程。十是工业企业基本建设工程。完成长恒药业厂房等14项工程建设。打好市区治理"十乱"战役（乱摆乱放、乱泼乱倒、乱贴乱画、乱挖乱占、乱搭乱建）。各县（市）完成48项工程，大安市完成青少年活动中心、体育场、交警指挥中心等工程；洮南市完成道路、绿化、步行街等工程；镇赉县完成房地产开发、亮化、文明通道等工程；通榆县完成集中供热、供水管网、停车场等工程。继续抓好全市交通、通讯设施建设，进一步改变白城面貌。

　　（七）加强精神文明建设，推动社会全面进步

　　认真贯彻公民道德建设实施纲要，弘扬爱国主义精神，以为人民服务为核心、以集体主义为原则、以诚实守信为重点，加强社会公德、职业道德和家庭美德教育。广泛开展文明城市、文明村镇、文明行业"等群众性精神文明创建活动，抓好"安白百里文明长廊"建设，提高城乡文明程度。加快科技创新，积极组织申报国家、省重大科技攻关项目，实施好中小企业创新项目，提高科技的贡献率。巩固教育改革和结构调整成果，抓好义务教育、职业教育和素质教育，扩大高中办学规模，加强农村薄弱学校改造和信息化建设。大力发展文化事业，培育文化产业，抓好社区和农村文化建设，办好"草原之夏"等系列文化活动。贯彻落实全民健身计划纲要，组织开展好群众性体育活动，促进竞技体育和群众体育协调发展。积极开展爱国卫生运动，努力创建卫生城。抓好计划生育工作，确保完成全年人口计划。充分发挥广播电视的舆论宣传作用，实施精品战略，提高宣传质量和档次，营造良好的舆论氛围。组织好双拥共建活动和国防后备力量建设，做好民族、宗教、侨务、地方志、防震减灾、人工降雨、人防、老龄、妇女儿童、残联、红十字会等工作，促进社会各项事业全面发展。

　　（八）着力化解社会矛盾，积极维护稳定大局

　　完成改革和发展的繁重任务，需要和谐稳定的社会环境。我们要充分运用经济、行政和法律手段，妥善处理和解决社会矛盾，保持安定团结的局面。

　　就业是民生之本。就业和再就业工作要以服务业为主攻方向，以非公有制经济为突破口，以中小企业为重点领域，努力创造就业岗位，开辟就业渠道，鼓励和扶持下岗失业人员自谋职业、自主创业。进一步完善劳动就业服务组织体系，建立健全公共就业服务制度。

　　抓好社会保障体系建设，扩大养老保险省级统筹、集体统筹和失业保险覆盖面，多方筹措资金，加大基金征缴和清欠力度，确保离退休和失业人员养老金、失业金的及时发放。抓好基本生活保障、失业保险和城市居民最低生活保障“三条保障线”工作，使符合条件的失业人员全部领到失业保险金，符合低保条件的全部纳入低保范围，确保他们的基本生活。抓好农村救灾救济，为弱势群体和生活困难群众多办实事、好事。

　　按照"分级负责、归口办理"原则，进一步落实信访工作领导责任制，完善领导接待日制度和信访责任追究制度，从源头上抓好信访工作，积极做好超前排查和矛盾化解，依法及时、就地解决信访问题。

　　深入开展"严打"整治斗争，依法严厉打击严重刑事犯罪和经济犯罪，推进治安防控体系建设；坚决打击"法轮功"等邪教组织的破坏活动，保持稳定的政治环境和良好的社会秩序。

　　坚持"安全第一，预防为主"的方针，严格执行安全生产责任制和责任追究制，继续开展安全生产的专项整治，完善预警和应急处理工作机制及预案，加大对各类隐患的整改力度。加强消防、交通等安全管理，防止重大事故发生。

　　　　三、进一步加强和改进政府工作

　　新的形势和任务对政府工作提出了更高的要求，我们要认真贯彻"三个代表"重要思想，坚持为民执政、科学理政、依法行政、从严治政，加强和改进政府工作，履行好经济调节、市场监管、社会管理、公共服务职能，努力为经济社会发展营造良好环境。

　　深入学习贯彻党的十六大精神，增强加快白城发展的强大动力。党的十六大提出了新世纪新阶段党和国家的奋斗目标和行动纲领，对我国改革开放和社会主义现代化建设作出了全面部署，为我们指明了前进方向。深入学习、宣传、贯彻党的十六大精神，是当前和今后一个时期的首要政治任务。各级政府要率先垂范，带头学习好、领会好、贯彻好十六大精神，紧紧把握精神实质，提高班子科学判断形势的能力、驾驭市场经济的能力、应对复杂局面的能力。要把十六大精神贯彻到基层，组织机关、学校、企事业单位采取集中讨论、办培训班等多种形式，推动学习活动的深入开展。运用十六大精神指导实践，解决当前经济和社会发展中存在的突出问题。充分利用报刊、广播、电视等宣传媒体，全方位、多形式宣传十六大精神，营造浓厚的舆论氛围，把广大干部群众的思想和行动统一到十六大精神上来，开创各项工作的新局面。

　　进一步解放思想，保持与时俱进的精神状态。推进白城跨越式发展，全面建设小康社会必须坚持不懈地解放思想，自觉地把思想认识从那些不合时宜的观念、做法和体制的束缚中解放出来。要引导广大干部群众增强升位翻番意识、兴工富市意识、城镇经济意识、能人当家意识、致富光荣意识、走出白城意识，进一步树立起与十六大精神要求相适应的发展理念，坚持与时俱进、自我加压、奋发图强、富市裕民，加快小康社会建设进程；适应经济国际化的需要，用世界眼光思考问题，用开放性思维谋划发展，扬长避短，培育优势，提升白城竞争力；紧跟时代发展步伐，推进工业化、信息化、城市化进程，努力实现经济社会跨越式发展；倡导识整体、顾全局，自觉做到局部利益服从整体利益，确保政令畅通，把方方面面的力量凝聚到推进整体升位和跨越式发展上来，使加快发展成为全市上下的共同意志。

　　加强民主法制建设，坚持依法行政。自觉接受人大的依法监督和工作监督，认真执行人民代表大会及其常委会的决议、决定，定期报告工作。积极支持人民政协履行职能，自觉接受民主监督。认真办理人大代表建议、议案和政协委员提案。密切同各民主党派、工商联和人民团体的联系。完善深入了解民情、充分反映民意、广泛集中民智、切实珍惜民力的决策机制，建立健全社情民意反映制度、与群众利益密切相关的重大事项社会听证制度和专家咨询制度，推进决策科学化、民主化。积极实行“四五"普法规划，扎实开展法制宣传教育，提高公民的法制素质。落实行政执法责任制，加强执法监督，提高依法行政水平。

　　改进政府管理经济方式，营造良好发展环境。深化行政审批制度改革，对现有的行政审批项目继续进行清理，巩固已取得的成果；对保留的行政审批项目，严格按照规定的审批范围开展工作，规范运作程序，完善监督制约机制，提高行政审批效率。要抓住关键环节，解决重点问题，提高政务公开水平。加快市政务服务中心建设，按照部门职能做好相关准备工作，力争在明年下半年投入运行。各县（市）也要立足实际搞好政务服务中心建设。加强市长和县（市、区）长公开电话工作，面向群众、面向基层、面向社会提供服务，切实为民排忧解难。加大市场经济秩序整治力度，坚决打击各种欺诈、制售假冒伪劣商品等非法经营行为，保护人民群众生命财产安全和切身利益。加快政务信息化建设，要把电子政务作为今后一个时期信息化工作的重点，以政务信息化带动、促进企业和社会信息化。继续抓“三网一库”建设，全面提升政府系统现有公用主干网络功能。加快建设综合化、智能化的宽带信息网，加强办公业务信息化，扩大党政机关、企事业单位联网面。搞好数字化示范小区的启动，努力提高信息化水平。

　　转变机关作风，促进工作落实。坚持深入实际调查研究，广泛听取基层的意见和建议，密切同人民群众的联系。对事关全局的重点工作要精心组织，靠前指挥，切实解决实际问题。加强督促检查，健全检查考核制度和考核评价体系，根据工作目标和责任制内容，及时督查落实。认真贯彻执行反腐倡廉的各项规定，全面落实廉政建设责任制，积极开展经济监督和领导干部经济责任审计工作，努力从源头上预防和治理腐败，用廉政建设和反腐败斗争的实际成果取信于民。

　　各位代表，全市经济和社会已进入新的历史发展时期，时代赋予了我们光荣而艰巨的使命。我们要认真贯彻落实党的十六大精神，在市委的领导下，高举旗帜，与时俱进，奋发图强，求真务实，全面完成各项任务，为推进白城整体升位和跨越式发展而努力奋斗！
